# Model-checking quantum systems

**DOI:** 10.1093/nsr/nwy106

**Published:** 2018-10-12

**Authors:** Mingsheng Ying, Yuan Feng

**Affiliations:** 1 Centre for Quantum Software and Information, University of Technology Sydney, Australia; 2 State Key Laboratory of Computer Science, Institute of Software, Chinese Academy of Sciences, China; 3 Department of Computer Science and Technology, Tsinghua University, China

## INTRODUCTION

We are currently in the midst of a second quantum revolution: *transition from quantum theory to quantum engineering* (e.g. quantum computing, communication, sensing). The main purpose of quantum theory is to find fundamental rules governing the existing physical systems. In contrast, quantum engineering aims at designing and implementing new systems (machines, devices, etc.) to achieve some desirable tasks, based on quantum theory.

From experience in today's engineering, it is not always easy for a human designer to completely understand the behaviours of the system that she/he is designing, and an error in her/his design may cause serious problems and even disasters. Consequently, theories and methodologies for verification of correctness, safety and reliability of complex engineering systems have been systematically studied in various engineering fields. In particular, computer scientists have developed techniques to verify the correctness of both hardware and software as well as the security of communication protocols.

### Second quantum revolution requires new verification techniques

Human intuition is poorly adapted to the quantum world compared to the classical world, which implies that human engineers will make many more mistakes in designing and implementing complex quantum systems such as quantum computer hardware and software and communication protocols. Even worse, because of the essential differences between the classical and quantum worlds, verification techniques for classical systems cannot be directly used for quantum systems. Novel verification techniques will be indispensable for the coming era of quantum engineering and technology.

### Model-checking techniques for classical systems

Model-checking is an effective technique to check whether a system satisfies a desired property. The properties that are checked are usually specified in a temporal logic; typical properties are deadlock freedom, invariants, safety, and request–response properties. The systems being checked are mathematically modelled as e.g. (finite-state) automata, transition systems, Markov chains and Markov decision processes [1].

In the last three decades, model-checking has become one of the dominant techniques for verification of computer hardware and software, and has proved mature as witnessed by a large number of successful industrial applications. Techniques of model-checking were even applied in systems biology recently.

With the emergence of quantum engineering and quantum technology, a question then naturally arises: *is it possible to*

*apply model-checking techniques in verifying the correctness and safety of quantum engineering systems, and how can this be done*?

### Difficulty in model-checking quantum systems

Unfortunately, due to some essential differences between classical and quantum systems, it seems unlikely that the classical model-checking techniques can be directly applied to quantum systems. Basically, to make model-checking techniques effective for quantum systems, the following three problems must be systematically addressed:

- *System modelling and property specification*: Behaviours of quantum systems cannot be described using classical modelling methods and, consequently, the properties of the quantum systems to be checked cannot be formalized by classical specification languages. As a result, novel conceptual frameworks must be proposed to properly model and consider quantum systems, including *formal models* and *formal descriptions of temporal properties* of quantum systems.
- *Quantum measurements*: Model-checking is usually applied to check the long-term behaviours of systems. However, to check whether a quantum system satisfies a certain property at a time point, one has to perform a quantum measurement on the system, which can change the state of the system. This makes studying the long-term behaviours of quantum systems much harder than with classical systems.
- *Algorithms*: Classical model-checking algorithms normally assume the state spaces to be finite or countably infinite. However, state spaces of quantum systems are inherently continuous. To develop algorithms for model-checking quantum systems, deep mathematical properties of the systems have to be exploited, so that a finite (or countably infinite) number of representative elements in the state spaces will suffice. Note that the state space of any quantum system has a natural linear algebraic structure. A well developed algorithm for verifying quantum systems should make clever use of this structure.

## EARLY RESEARCH ON MODEL-CHECKING OF QUANTUM SYSTEMS

Despite the difficulties discussed above, a few model-checking techniques for quantum systems have been developed in the last 10 years. The earliest work mainly targeted checking quantum communication protocols:

- Taking the probabilism arising from quantum measurements into account, the probabilistic model-checker PRISM was used in [2] to verify the correctness of quantum protocols, including superdense coding, quantum teleportation and quantum error correction.
- A branching-time temporal extension of exogenous quantum propositional logic was introduced and then the model-checking problem for this logic was studied in [3], with verification of the correctness of quantum key distribution BB84 as an application.
- A linear temporal extension of exogenous quantum propositional logic was then defined and the corresponding model-checking problem was investigated in [4].
- Model-checking techniques were developed in [5] for quantum communication protocols modelled in process algebra CQP (communicating quantum processes) [6].
- A model-checker for quantum communication protocols was also developed in [7], where only the protocols that can be modelled as quantum circuits expressible in the stabilizer formalism were considered. This technique was further extended beyond stabilizer states and used to check equivalence of quantum protocols.

## MODEL-CHECKING QUANTUM AUTOMATA

A research line pursued by the authors and their collaborators is to develop model-checking techniques that can be used not only for quantum communication protocols but also for general quantum systems, including physical systems and quantum programs.

Quantum automata were adopted in [8,9] as the model of the systems:

Definition 1(Quantum automata [10]). A quantum automaton is a 4-tuple $\mathcal {A}=(\mathcal {H},\mathit {Act}, \lbrace U_\alpha :\alpha \in \mathit {Act}\rbrace ,\mathcal {H}_0)$, where:

1) $\mathcal {H}$ is a finite-dimensional Hilbert space, called the state space;
2) $\mathit {Act}$ is a finite set of action names;
3) for each action name $\alpha \in \mathit {Act}$, *U*_α_ is a unitary operator on $\mathcal {H}$;
4) $\mathcal {H}_0\subseteq \mathcal {H}$ is the subspace of initial states.

A quantum automaton behaves as follows: it starts from some initial state in $\mathcal {H}_0$, and at each step it performs a unitary transformation *U*_α_ for some $\alpha \in \mathit {Act}$. An algorithm for checking certain linear-time properties (e.g. invariants and safety properties) was proposed in [8], where, following Birkhoff–von Neumann quantum logic, closed subspaces of the state Hilbert space are used as the atomic propositions about the state of the system, and the checked linear-time properties are defined as infinite sequences of sets of atomic propositions. Furthermore, the decidability or undecidability of several reachability problems for quantum automata were established in [9].

## MODEL-CHECKING QUANTUM MARKOV CHAINS

The model-checking problem for a larger class of quantum systems than quantum automata, namely quantum Markov chains, was studied in [11].

Note that continuous-time quantum Markov processes have been studied intensively in mathematical physics. Discrete-time quantum Markov chains were recently introduced as a semantic model for quantum programs.

Definition 2 (Quantum Markov chains [11]). A quantum Markov chain is a triple $(\mathcal {H},\mathcal {E},\mathcal {H}_0)$, where $\mathcal {H}$ and $\mathcal {H}_0$ are the same as in Definition 1, and $\mathcal {E}$ is a super-operator on $\mathcal {H}$.

A quantum Markov chain starts in an initial state in $\mathcal {H}_0$, and at each step it performs the (same) quantum operation modelled by the super-operator $\mathcal {E}$. Note that the (discrete-time) dynamics of closed quantum systems is usually depicted by unitary operators, and the behaviours of open quantum systems are described by super-operators. Obviously, the notion of quantum automata can be generalized by replacing unitary operators *U*_α_ in Definition 1 by super-operators $\mathcal {E}_\alpha$. Furthermore, quantum Markov decision processes [12] can be defined by introducing decision strategies into such generalized quantum automata.

Several algorithms for checking the reachability of quantum Markov chains and quantum Markov decision processes have been developed. As in checking classical Markov chains and Markov decision processes, graph reachability is a key to these algorithms. However, classical graph theory is not suited to our purpose; instead, a new theory of quantum graphs (i.e. graphs in a Hilbert space with an adjacency relation induced by a super-operator) was developed and, in particular, an algorithm for the BSCC (bottom strongly connected components) decomposition of the state Hilbert spaces was found in [11]. Another decomposition technique, namely periodic decomposition, for quantum Markov chains has recently been proposed.

## MODEL-CHECKING SUPER-OPERATOR-VALUED MARKOV CHAINS

The notion of the super-operator-valued Markov chain is introduced in [13] as a higher-level model of quantum programs and quantum cryptographic protocols.

Definition 3 (Super-operator-valued Markov chains [13]). A labelled super-operator-valued Markov chain over a set *AP* of predefined atomic propositions is a 5-tuple $(S, s_0, \mathcal {H}, Q, L)$, where:

1) *S* is a finite set of *classical states* with *s*_0_ ∈ *S* being the initial state;
2) $\mathcal {H}$ is a finite-dimensional Hilbert space, called the quantum state space;
3) $Q\! : S\times S\rightarrow \mathcal {SO}_\mathcal {H}$ is a transition super-operator function, where $\mathcal {SO}_\mathcal {H}$ denotes the set of trace-nonincreasing super-operators on $\mathcal {H}$ and, for each *s* ∈ *S*, ∑_*t* ∈ *S*_*Q*(*s*, *t*) is trace-preserving; and
4) *L*: *S* → 2^*AP*^ is a labelling function.

A super-operator-valued Markov chain has two state spaces, a classical one and a quantum one, which are connected through the transition super-operator function. It behaves in a similar manner to classical Markov chains. It starts from the classical initial state *s*_0_ but with the quantum initial state unspecified (it can be taken arbitrarily). Then at each step, given the current classical state *s* and quantum state ρ, it proceeds to a classical state *t* with probability tr[*Q*(*s*, *t*)(ρ)], and the accompanying quantum state evolves into *Q*(*s*, *t*)(ρ)/tr[*Q*(*s*, *t*)(ρ)] provided that tr[*Q*(*s*, *t*)(ρ)] ≠ 0. The normalization requirement that ∑_*t* ∈ *S*_*Q*(*s*, *t*) is trace-preserving guarantees that the probabilities of going from *s* to some classical state sum up to 1.

As the atomic propositions are taken to be classical (they apply only to classical states), this Markov chain model is suitable for verification of quantum systems against classical properties, such as running time, termination, reachability, etc. One distinct feature of this model, however, is that it allows us to check the properties of the system *once and for all*; i.e. the verified results apply to all initial quantum states. For example, the model-checking algorithm for the reachability problem essentially calculates a positive operator Π, accounting for all (classical) paths satisfying the concerned property. Then the reachability *probability* when the Markov chain starts in the initial quantum state ρ is simply tr(Πρ).

A corresponding computation tree logic (CTL) for super-operator-valued Markov chains was defined, and algorithms for checking such properties were developed in [13]. A tool implementation of these algorithms has been provided [14] based on a probabilistic model-checker. Algorithms for model-checking ω-regular properties, a general class of properties subsuming linear temporal logic (LTL) formulas, against super-operator-valued Markov chains have been proposed [15], thus allowing analysis of a wide range of properties such as repeated reachability, reachability in a restricted order, and nested Until properties. Furthermore, the reachability problem of a recursive extension of super-operator-valued Markov chains was studied in [16], with the application of analysing quantum programs with procedure calls.

## CONCLUSION

As reviewed in previous sections, several theoretical frameworks and algorithms of quantum model-checking have been developed. Certainly, however, quantum model-checking is still at a very early stage of its development; in particular, its applications are only at the level of toy examples. We envisage that, in the future, quantum model-checking techniques can be applied to the following areas:

1. *Checking physical systems*: Physicists have already considered the algorithmic checking problem of certain properties of quantum systems, e.g. quantum measurement occurrence [17] and reachability of quantum states [18]. Quantum model-checking can offer a systematic view of this line of research.
2. *Verification of quantum circuits*: Verification of circuits has been one of the major application areas of classical model-checking. But model-checking applied to verification of quantum circuits is an area to be systematically exploited.
3. *Analysis and verification of quantum programs*: Another important application area of classical model-checking is analysis and verification of programs. Several techniques for analysis and verification of quantum programs have been reported in the last few years [19,20]. However, model-checking techniques specifically designed for quantum programs are still missing.
4. *Verification of security of quantum communication protocols*: Applications of model-checking mentioned in the section entitled ‘Early research on model-checking of quantum systems' focus on verification of the correctness of quantum communication protocols. However, verification of the security of quantum protocols is much more difficult, and model-checking applied to it is an interesting topic for future research.

## References

[1] Baier C , Katoen JP. Principles of Model Checking. Cambridge: MIT Press, 2008.

[2] Gay SJ , Nagarajan R, Papanikolaou N. arXiv:quant-ph/0504007.

[3] Baltazar P , Chadha R, Mateus P. Int J Quant Inform 2008; 6: 219–36.

[4] Mateus P , Ramos J, Sernadas A Temporal logics for reasoning about quantum systems. In: Mackie I, Gay S (eds). Semantic Techniques in Quantum Computation. Cambridge: Cambridge University Press 2009; 389–413.

[5] Davidson T , Gay SJ, Mlnarik H Int J Unconv Comput 2012; 8: 73–98.

[6] Gay SJ , Nagarajan R. Communicating quantum processes. In: Abadi M (ed.). Proceedings of the 32nd ACM Symposium on Principles of Programming Languages (POPL). New York: ACM Press, 2005, 145–57.

[7] Gay SJ , Nagarajan R, Papanikolaou N. QMC: a model checker for quantum systems. In: Gupta A and Malik S (eds). Proceedings of the 20th International Conference on Automated Verification (CAV'08), Lecture Notes in Computer Science 5123. Berlin: Springer, 2008, 543–7.

[8] Ying MS , Li YJ, Yu NK ACM Trans Comput Logic 2014; 15: 22.

[9] Li YJ , Ying MS. (Un)decidable problems about reachability of quantum systems. In: Baldan P (ed.). Proceedings of the Concur. Berlin: Springer, 2014, 482–96.

[10] Kondacs A , Watrous J. On the power of quantum finite state automata. In: Proc. 38th Symposium on Foundation of Computer Science (FOCS'97). Los Alamitos, California: IEEE Press, 1997, 66–75.

[11] Ying SG , Feng Y, Yu NK Reachability probabilities of quantum Markov chains. In: D'Argenio, PR and Melgratti H (eds). Proceedings of Concur'13. Berlin: Springer, 2013, 334–48.

[12] Barry J , Barry DT, Aaronson S. Phys Rev A 2014; 90: 032311.

[13] Feng Y , Yu NK, Ying MS. J Comput Syst Sci 2013; 79: 1181–98.

[14] Feng Y , Hahn EM, Turrini A QPMC: A model checker for quantum programs and protocols. In: Bjørner N and de Boer F (eds). FM'15: Lecture Notes in Computer Science Vol. 9109. Berlin: Springer, 2015, 265–72.

[15] Feng Y , Hahn EM, Turrini A Model checking omega-regular properties for quantum Markov chains. In: Meyer R and Nestmann U (eds). Proceedings of the Concur. Dagstuhl: LIPICS, 2017, 35: 1–16.

[16] Feng Y , Yu NK, Ying MS. Reachability analysis of recursive quantum Markov chains. In: Chatterjee K and Sgall J (eds). Proceedings of MFCS, Berlin: Springer, 2013, 385–96.

[17] Eisert J , Müller MP, Gogolin C. Phys Rev Lett 2012; 108: 260501. 23004944 10.1103/PhysRevLett.108.260501

[18] Schirmer SG , Solomon AI, Leahy JV. J Phys A: Math Gen 2002; 35: 8551–62.

[19] JavadiAbhari A , Patil S, Kudrow D Parallel Comput 2015; 45: 2–17.

[20] Ying MS . Foundations of Quantum Programming. Burlington: Morgan Kaufmann, 2016.

